# Supplementary material for: Intestinal Dominance by Serratia marcescens and Serratia ureilytica among Neonates in the Setting of an Outbreak
Source: Microorganisms. 2021 Oct 31;9(11):2271. doi: 10.3390/microorganisms9112271 (PMC8624583; doi:10.3390/microorganisms9112271)
Supplement: Supplementary file 1 [file microorganisms-09-02271-s001.zip › Supplementary Table S1.pdf]

Supplementary Table S1. Whole-genome sequencing parameters for the *de novo* assembly of the selected isolates.

Isolate	Number of Reads	Genome Length	Depth	Number of Contigs	N50	GC Content
**S1**	1,111,802	5,907,428	28.2×	1,381	39,844	59.4%
**S3B**	1,765,530	5,298,447	48.4×	57	349,653	59.4%
**S9**	1,092,178	5,298,228	30.5×	57	200,422	59.4%
**S17**	1,154,034	4,615,693	27.3×	272	29,860	59.5%
**S18**	1,876,700	4,893,882	64.0×	32	402,773	59.5%
**S19**	4,000,000	4,895,847	131.3×	31	362,707	59.5%
