# Supplementary figures and images for: Intestinal Dominance by Serratia marcescens and Serratia ureilytica among Neonates in the Setting of an Outbreak
Source: Microorganisms. 2021 Oct 31;9(11):2271. doi: 10.3390/microorganisms9112271 (PMC8624583; doi:10.3390/microorganisms9112271)

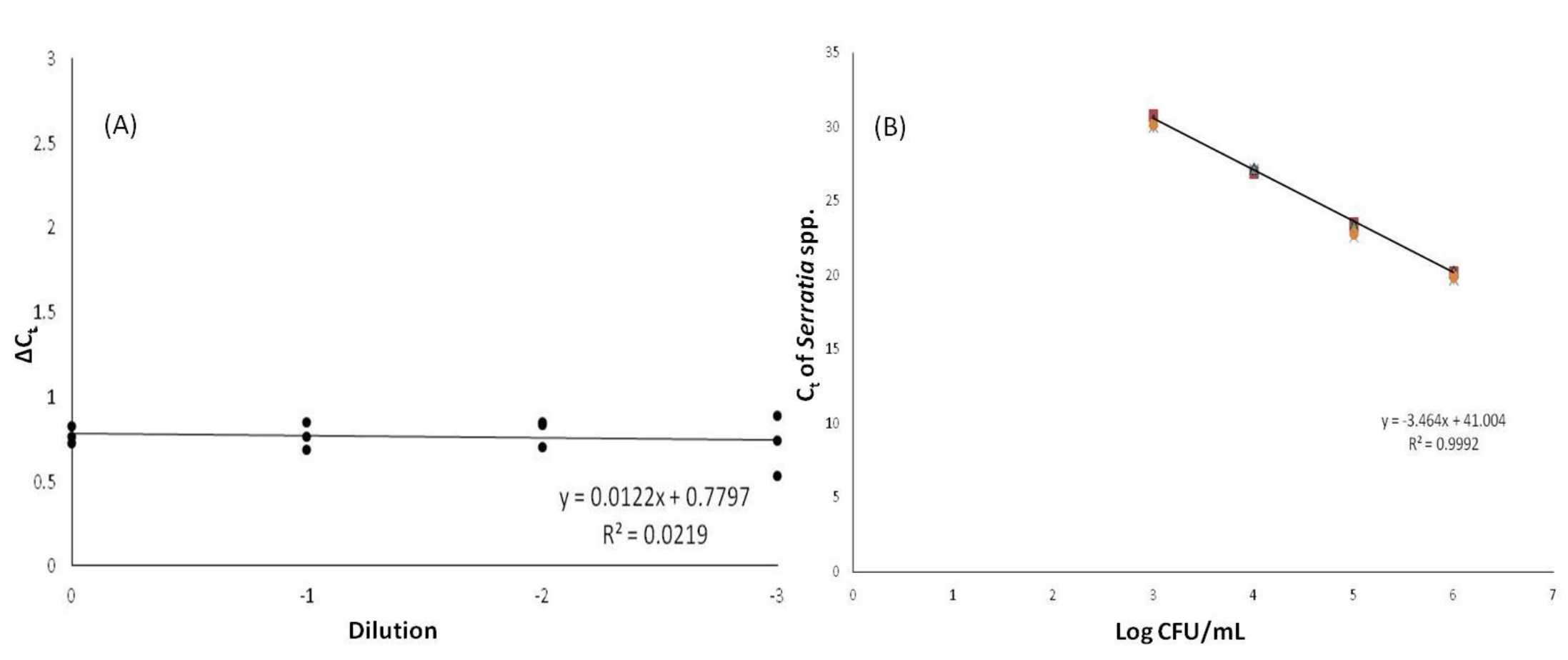

Supplement: Supplementary file 1 [file microorganisms-09-02271-s001.zip › Supplementary Figure S1.jpg]

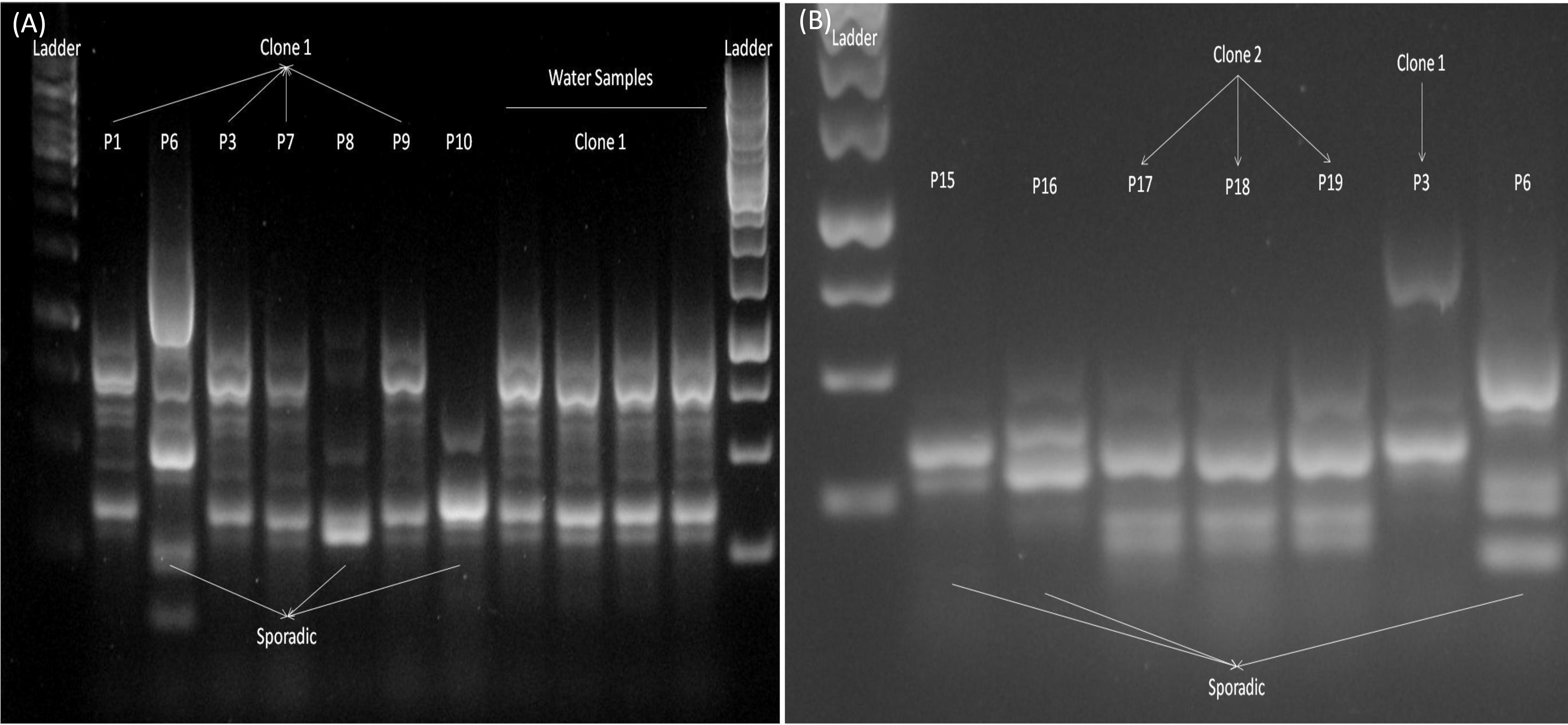

Supplement: Supplementary file 1 [file microorganisms-09-02271-s001.zip › Supplemetary Figure S2.jpg]

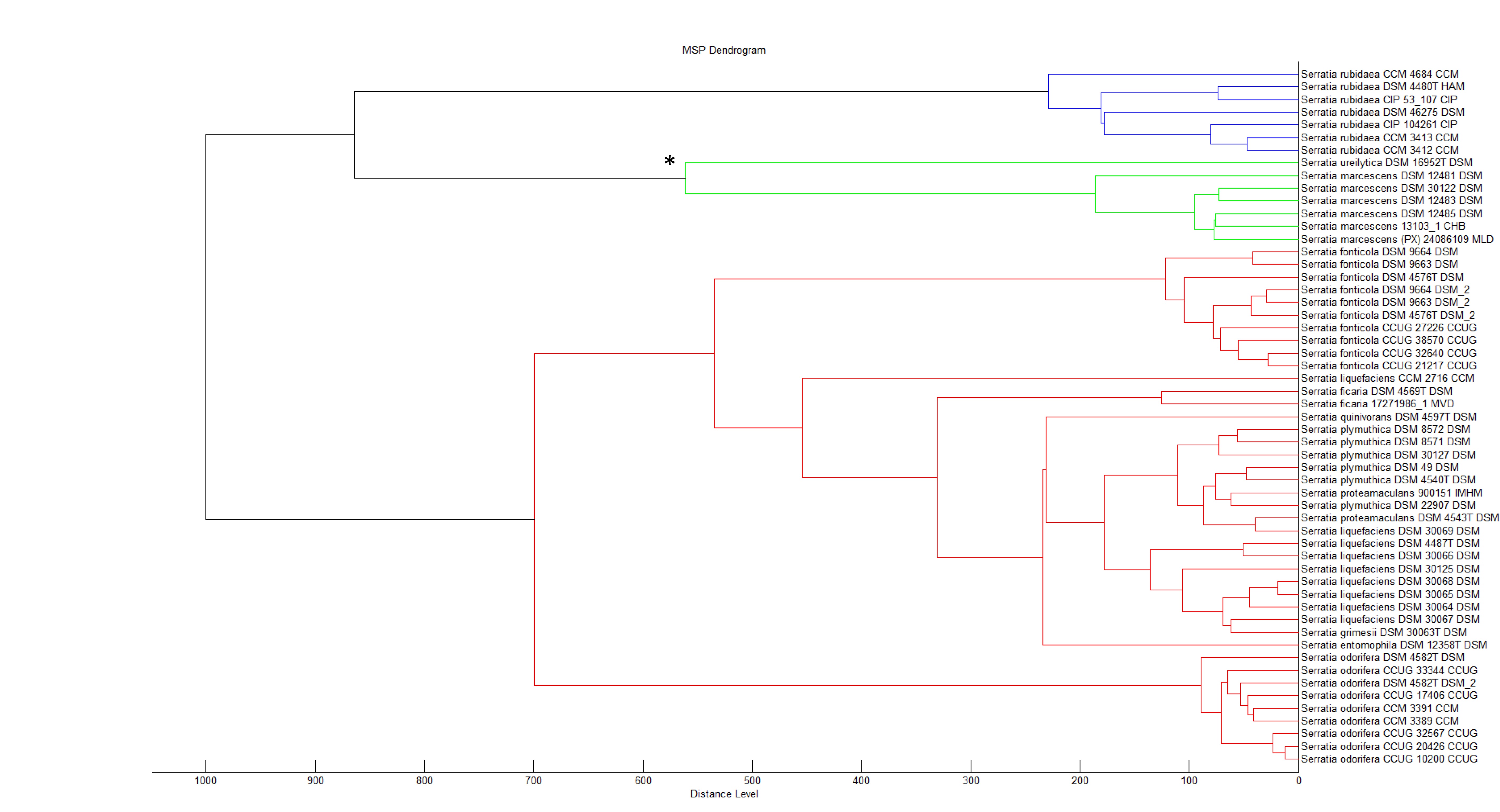

Supplement: Supplementary file 1 [file microorganisms-09-02271-s001.zip › Supplemetary Figure S3.jpg]
